# Lateral trochanteric bursa repair improves early hip function after posterior approach total hip arthroplasty: a prospective randomized control trial

**DOI:** 10.1186/s42836-022-00127-6

**Published:** 2022-06-06

**Authors:** Byron E. Chalidis, Nick P. Sachinis, Gabrielle Hawdon, Stephen McMahon

**Affiliations:** Malabar Orthopaedic Clinic, 43 The Avenue, Windsor, Melbourne, 3181 Australia

**Keywords:** Total hip arthroplasty, Trochanteric bursa, Trochanteric pain, Thigh pain, Harris hip score, Hip function

## Abstract

**Background:**

The trochanteric bursa (TB) is an anatomic structure, which is dissected during posterior/lateral hip approaches in Total Hip Arthroplasty (THA). Some surgeons prefer to simply resect the bursa as they believe that it may be responsible for postoperative lateral trochanteric pain (LTP). Others advocate that this tissue acts as a buffer minimizing friction between soft tissue and bone, and therefore its repair may prevent LTP after THA.

**Aim:**

The purpose of this prospective randomized controlled trial was to compare the clinical results of either resecting or repairing the TB during posterior approach THA.

**Methods:**

Forty-two patients with primary hip osteoarthritis undergoing THA via a posterior hip approach were randomly assigned to two groups; Group A, or TB resection group and Group B, or TB repair group. All patients in both groups were evaluated postoperatively in terms of hip function, measured by the Harris Hip Score (HHS), at 6 weeks, 3 months, 6 months, and 12 months after surgery, as well as LTP during daily routine activities and lying on the operative side.

**Results:**

Forty patients completed the study. Postoperative difference in terms of leg length and femoral offset was similar among the two groups (*P* = 0.467 and *P* = 0.39, respectively). At 6 weeks, patients in Group B had higher HHS (*P* = 0.052) and experienced less LTP when lying on the operative side (*P* = 0.046) but not during activities (*P* = 0.759). Thereafter, all functional parameters measured had comparable values in both groups. Subgroup analysis failed to identify any correlation between high offset stems and LTP.

**Conclusion:**

TB repair in posterior approach THA improves hip functional recovery as well as patients’ ability to lie on the operative side during the early postoperative period.

## Introduction

The trochanteric bursa (TB) is a complex anatomic structure that has been thoroughly examined in anatomical and radiological studies [1–3]. Macroscopically, this large fluid-filled sac is centred over the lateral surface of the greater trochanter and it is separated from the bone only by the distal tendon of gluteus medius and the origins of vastus lateralis, which it partially covers. However, in contrast with many standard descriptions, two or more bursae potentially may be present and arranged in different layers close to the apex of the greater trochanter. The dominant, deep subgluteus maximus bursa, which is normally considered the “trochanteric bursa”, can be accompanied by either a superficial and/or secondary deep subgluteus maximus bursa and a distal gluteofemoral bursa [3].

The variable anatomy gave rise to the hypothesis that bursa development might be acquired as a consequence of excessive friction between the greater trochanter and the gluteus maximus as it inserts into the fascia lata [1]. Furthermore, the consistent finding of one or more bursae around the trochanteric region suggests that this structure has a significant function. It facilitates the gliding and smooth motion of the gluteal tendons, iliotibial band, and tensor fascia lata over the greater trochanter. The presence of multiple small blood vessels on the external surface of the bursa indicates increased vascularity to the area that may further improve nutrition and intrinsic healing capacity of the attached abductors tendons [1].

The trochanteric bursa is usually divided or resected during the posterolateral hip approach in Total Hip Arthroplasty (THA). The development of lateral trochanteric pain (LTP) after THA can be problematic for the patient, impacting daily activities and disturbing sleep. This problem seems to be multifactorial and a variety of surgical parameters, patient-related factors and biomechanical alterations after THA have been associated with the development of pain around the trochanteric area. The lack of definitive evidence has led to diverse opinions regarding the optimal intraoperative management of the TB. Some surgeons prefer to simply resect the bursa as they believe that it may be responsible for postoperative LTP. Others advocate that preservation of the bursal tissue lateral to the greater trochanter may add a “protection” layer to the hip joint and minimize friction between the soft tissue and the bone interface.

So far, little attention has been given to the management of the TB during THA. We are unaware of any relevant studies addressing this topic. The aim of this randomized study was to investigate the effect of resecting or repairing the TB in patients undergoing posterior-approach THA. Particularly, we focused on hip function and development of LTP at different postoperative time points.

## Methods

### Study design and population

This was a single-centre, prospective randomized, two-arm, equivalence clinical trial that was approved by the Avenue Hospital Human Research Ethics Committee (Ethics Approval Number 104) and was registered with the Australian and New Zealand Clinical Trials Registry (registration number ACTRN12609000355279) prior to recruitment of patients. The study complied with the National Health and Medical Research Council’s (NHMRC) National Statement on Ethical Conduct in Human Research (2007).

**Fig. 1:**
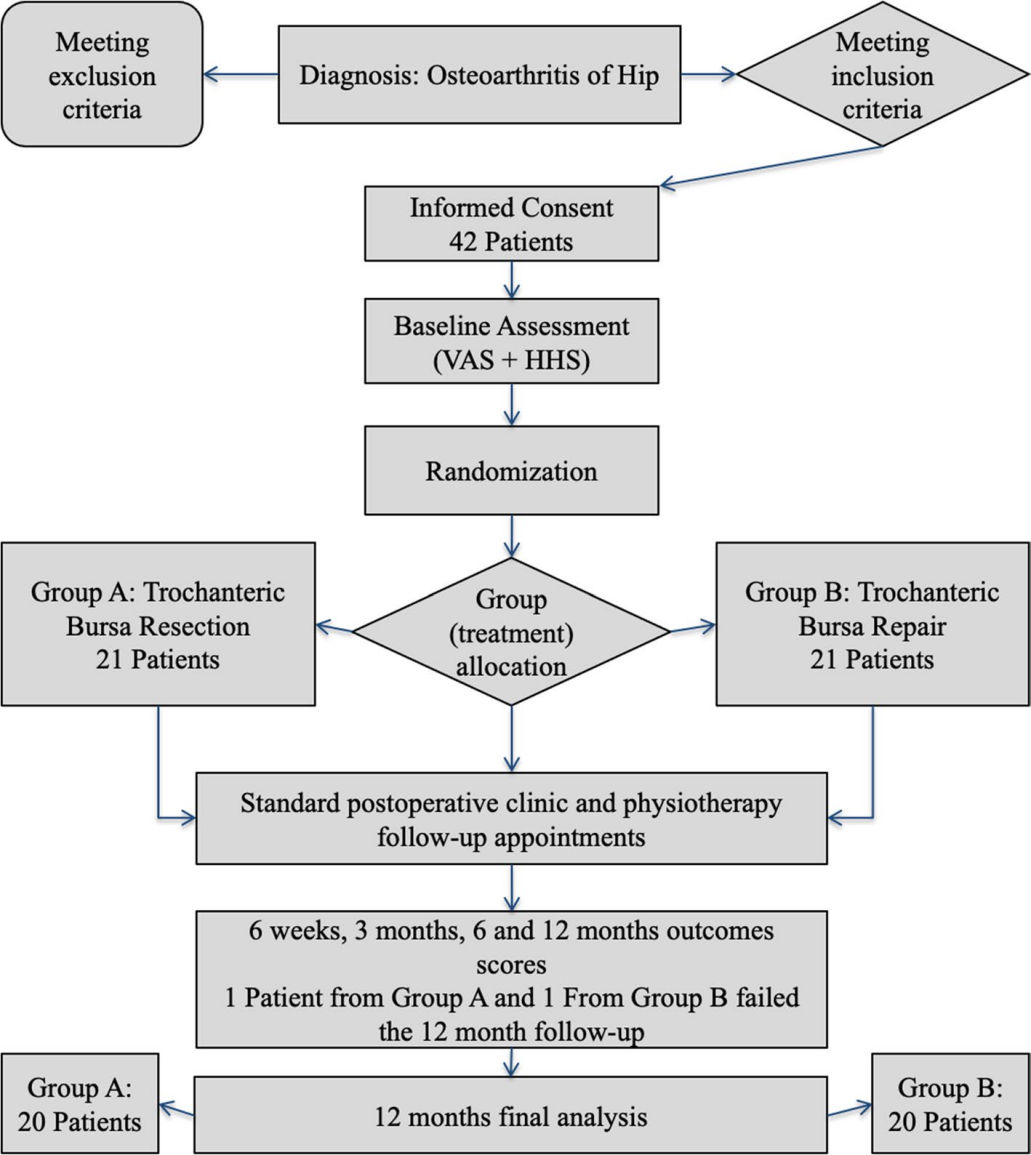
Flow diagram (CONSORT) of study. VAS: Visual Analogue Scale; HSS: Harris Hip Score

Forty-two patients (ASA I-II) with primary hip osteoarthritis were recruited and underwent a primary THA procedure. Exclusion criteria were: age < 40 or > 75 years; previous surgical interventions in the ipsilateral hip and thigh; documented hip abductor tendons pathology; previous corticosteroid injection at trochanteric area at least 1 year before THA; significant hip joint bone defect; leg length discrepancy of more than 1 cm; chronic low back pain; malignancy; inflammatory arthritis; metabolic diseases; severe bone and musculoskeletal diseases. Figure 1 depicts the flow diagram (CONSORT) of the study.

All enrolled patients provided written consent for the study. Electronic health records were accessed and provided the baseline demographic and medical history data which are summarized in Table 1.

**Table 1:** Demographic preoperative data of study. Age, Visual Analogue Scale (VAS) and Harris Hip Score (HHS) are presented by means and standard deviation. For gender, ASA anaesthesiologic status and operative side, the absolute number is used

Variable (Mean ± SD)	Group A (Bursa resection)	Group B (Bursa repair)	*P*-Value
**Age (years)**	62.4 ± 9.1	63.8 ± 8.8	0.565
**Gender (Male/Female)**	7/13	8/12	0.744
**ASA I/II**	12/8	14/6	0.741
**Operative side (Right/Left)**	11/9	12/8	0.749
**Preoperative VAS**	4.9 ± 1.52	5.35 ± 1.42	0.340
**Preoperative HHS**	53.8 ± 11	51.6 ± 14	0.583

### Patient allocation and randomisation

Patients were randomized to undergo resection of the TB (Group A) or repair (Group B) in a 1:1 ratio, by using a computerized randomization system at the time of consent. These patients were also given a specific code (*e*.*g*. Patient number 123). We used the RAND function of the Office Excel program (Microsoft). We created 42 cells with numbers in them, and rounded them up so that they had no decimals. Odd numbers constituted the trial group, and even numbers constituted the control group. The RAND function was run a few times until an equal quantity of even and odd numbers had been created. Patients were then placed in consecutive cells after recruitment and were randomized accordingly. The surgeon’s secretary was unblinded and created a note for each patient. This note was placed inside an opaque envelope. The individuals performing the randomization process and producing the blinded data sheets (with patients’ codes instead of names), were not involved in the operation or the follow-ups. Participating surgeons were notified at the time of surgery via the sealed envelopes, which accompanied the patient. Research personnel performing the outcome assessments at regularly scheduled orthopedic clinic visits were also blinded to subject allocation.

During the trial, one male patient from Group A moved residence and was unable to complete the final follow-up. Also, one female patient from Group B did not attend the latest review for unknown reasons. Neither of these patients had any complications reported up to 6-month follow-up. Due to the small dropout percentage and statistical indifference that resulted from this follow-up loss, the authors of this study decided to analyze the results of the remaining 40 patients, 20 for each group.

### Trial procedure

All operations were performed by one senior specialized orthopedic surgeon and one fellowship-trained surgeon. All patients received a posterior approach THA under general or spinal anesthesia. Trochanteric bursa was evident in all cases and was amenable to later repair. Routine preparation of acetabulum and proximal femur was performed and a cementless THA was performed using a hemispherical porous-coated acetabular cementless cup (R3, Smith & Nephew Synergy, Memphis, Tennessee, USA) and a porous-coated tapered conical stem (Synergy®; Smith and Nephew, Memphis, TN, USA). Ceramic on ceramic bearing surfaces (Biolox Delta®; CeramTec, Plochingen, Germany) or a combination of a ceramic femoral head (Biolox Delta®; CeramTec, Plochingen, Germany) with a highly cross-linked polyethylene liner (Smith & Nephew Synergy, Memphis, Tennessee, USA) were selected according to patients’ age and physical status.

**Fig. 2:**
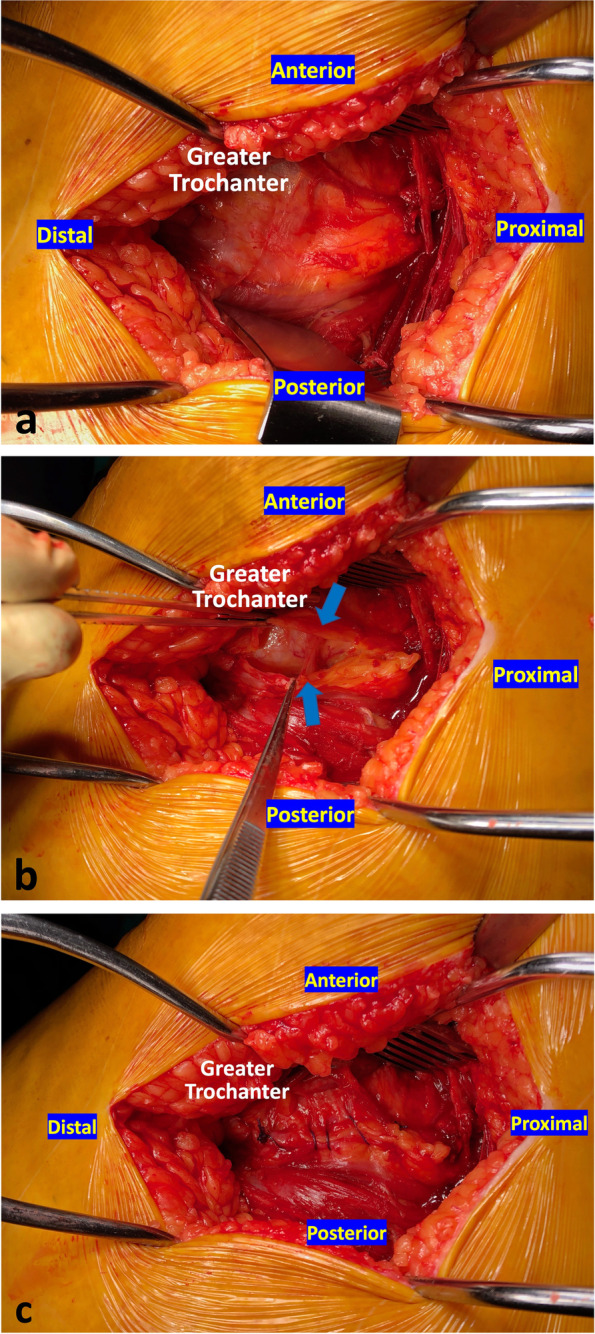
Intraoperative picture of the posterior hip approach (**a**); The trochanteric bursa (blue arrows) is incised (**b**); In case of bursa repair (Group B), this was performed by using a No 1 absorbable continuous suture (Vicryl Ethicon, Johnson & Johnson, Somerville, NJ) (**c**)

During the procedure, repair of the posterior capsule and short external rotators was performed using No 2 absorbable interrupted sutures (Vicryl Ethicon, Johnson & Johnson, Somerville, NJ). For the repair group, closure of the bursa was performed by using a No 1 absorbable continuous suture (Vicryl Ethicon, Johnson & Johnson, Somerville, NJ). The overlying fascia and subcutaneous fat tissues were re-approximated with No 2 and No 0 absorbable running stitches (Vicryl Ethicon, Johnson & Johnson, Somerville, NJ). Skin was closed via a continuous running technique, with 3–0 Monocryl subcuticular suture (Ethicon, Inc., Somerville, NJ) (Fig. 2).

Postoperatively, an identical rehabilitation protocol was followed for both groups and all patients were allowed to lie on the operative side 1 week after the operation and recorded any pain or discomfort. Follow-up was scheduled at 6 weeks, 3 months, 6 months and 12 months postoperatively.

### Study objectives

The first objective of the study was to analyze the clinical outcomes of either TB repair or resection during posterior hip approach in patients undergoing THA for primary osteoarthritis. This was assessed by using the following quantitative scoring systems before surgery and 6 weeks, 3 months, 6 months and 12 months after surgery:

- Harris Hip Score (HSS), in order to quantify the overall function of the operated hip. This was the main evaluation index of the trial.
- Visual Analogue Scale (VAS) score (1–10) for evaluation of LTP during daily normal daily activities (walking, sitting and climbing stairs) and lying on the operative side.

Postoperative screening and data collection were performed by an independent investigator (GH) who did not participate in the randomization process and operative procedures and was not aware of the method applied to patients.

Another objective of the study was to identify any difference between standard and high offset stems in terms of LTP. Alteration of femoral offset (perpendicular distance between the centre of rotation of femoral head and a line drawing through the centre of femoral canal) after surgery was also compared in both groups. In addition, any correlation between TB repair and operative and hospitalisation times was recorded.

### Data analysis

This trial was designed as an equivalence trial and the null hypothesis was that no statistically significant difference would arise from analysis of the clinical scores in both groups. Data were summarized using the mean, standard deviation and range for continuous variables, the absolute number for categorical variables, the median and range for non-parametric variables. The HSS was the primary outcome and the VAS for pain was the secondary outcome. Both variables were evaluated at specific pre- and postoperative time points, before surgery and 6 weeks, 3 months, 6 months and 12 months after surgery. The means of the two groups were compared using hypothesis testing. Continuous variables were evaluated for normal distribution using the Shapiro-Wilk test. Normally distributed variables were studied with the Student’s *t*-test. Categorical variables were assessed using Fisher’s exact test. The postoperative variables were not normally distributed and hypothesis testing was conducted using the Mann-Whitney test for HHS and the linear-by-linear association of the Chi-Square analysis. Sample size calculations were made with the G* power (v 3.1) program and utilized a power of 0.80 (type II error rate) and an alpha (type I error rate) of 0.05 [4]. Harris Hip Score was the main indicator for estimation of the sample size. For a two-tailed *t*-test, an estimated 10 point minimal clinically important difference of HHS and an effect size of 0.9, a total sample size of 42 patients was calculated. To the best of our knowledge, at the time of study design, no studies that provided conclusive data regarding the minimal clinically important difference on the HHS existed. Therefore, the numbers for the power calculation were estimated by the study team, after analyzing historic HHS data from patients of the local institution. All tests used a *P*-value < 0.05 as the criterion for statistical significance. All data were analyzed using SPSS statistical software (version 26, IBM, Armonk, NY, USA).

## Results

Demographic data were comparable in both groups (*P* > 0.05) (Table 1). No significant differences in terms of operative and hospitalisation time as well as intraoperative variables were identified between the two groups (Table 2). The mean difference in femoral offset was 1.96 ± 0.77 in Group A and 2.17 ± 0.75 in Group B without reaching statistical significance among both groups (*P* = 0.39).

**Table 2:** Clinical data comparison between groups. Operative and hospitalisation time are presented by means and standard deviation values. For type of anesthesia, CoP/CoC (Ceramic on Polyethylene/Ceramic on Ceramic) bearings, femoral head and offset type, absolute numbers were used. Leg length discrepancy is demonstrated via the median number with range in brackets

Variables (Mean ± SD)	Group A (Bursa resection)	Group B (Bursa repair)	***P***-Value
**Operative Time (min)**	133.5 ± 13	136.5 ± 9.6	0.411
**Hospitalisation Time (days)**	4.35 ± 1.49	4.15 ± 1.46	0.671
**General/Spinal Anesthesia**	13/7	14/6	0.736
**CoP/CoC Articulation**	15/5	17/3	0.677
**Femoral Head 32 mm/36 mm**	4/16	5/15	0.705
**Standard/High Offset Stem**	11/09	10/10	0.752
**Leg Length Discrepancy (mm)**	1 (0 to 5)	1 (0 to 4)	0.467

**Fig. 3:**
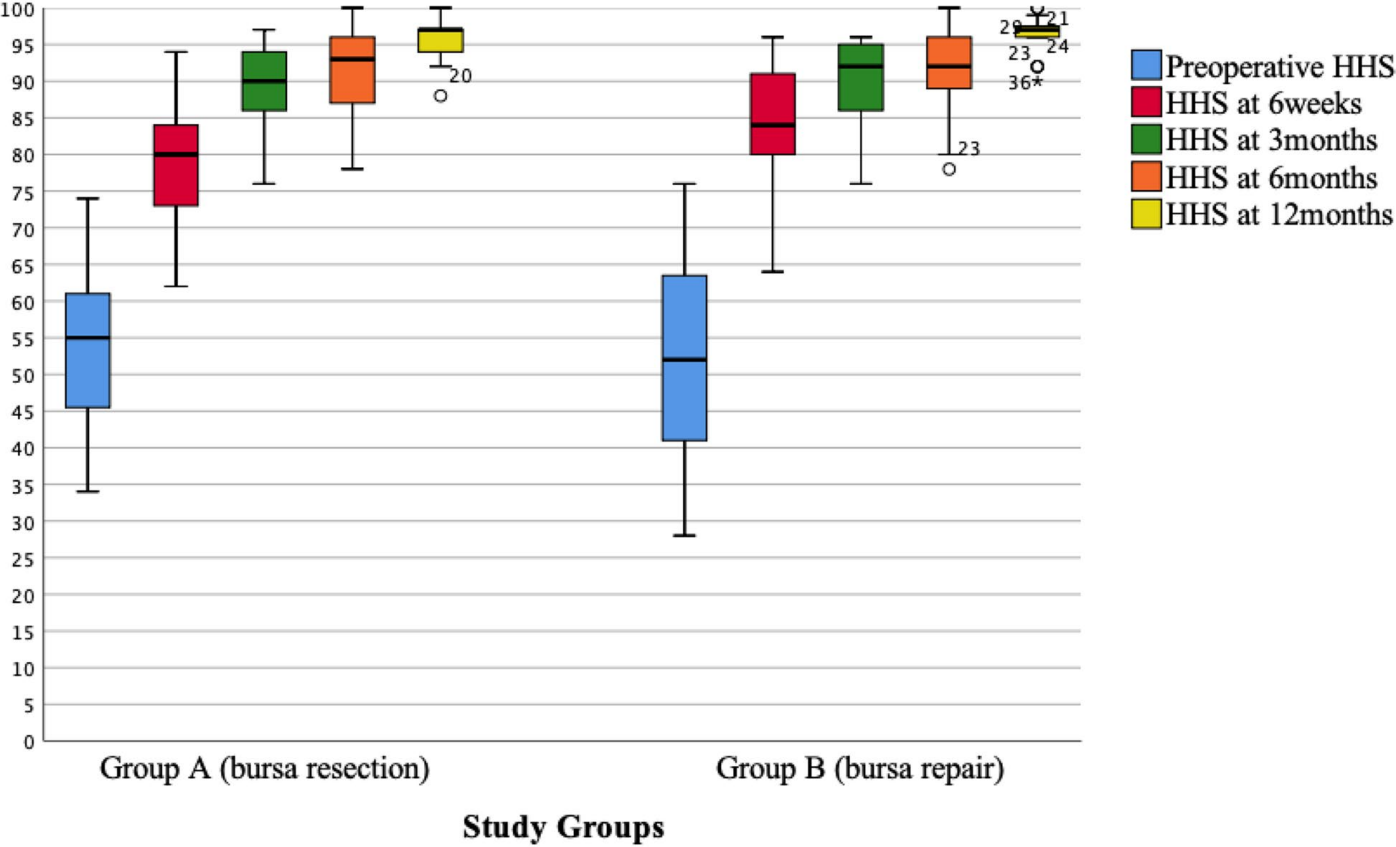
Box plots of Harris Hip Score (HHS) in both groups at all examined time points. Ends of boxes define the 25th and 75th percentiles, with line at median. Whiskers represent minimum and maximum values, respectively. Small circles and asterisks represent outliers.

**Fig. 4:**
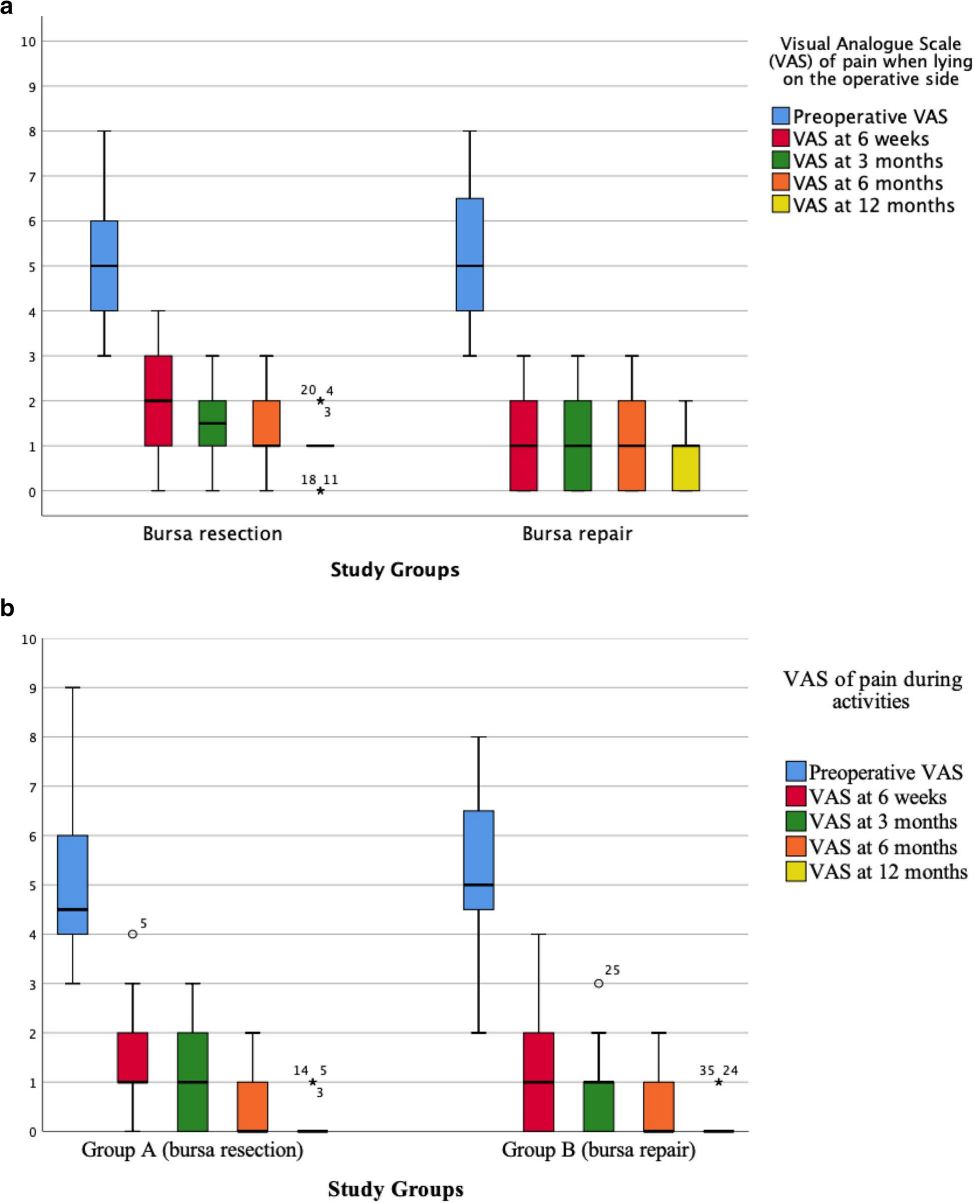
Box plots of Visual Analogue Scale (VAS) pain score throughout the study in both groups when lying on the operative side (**a**) and during activities (**b**). Ends of boxes define the 25th and 75th percentiles, with line at median. Whiskers represent minimum and maximum values, respectively. Small circles and asterisks represent outliers

At 6 weeks postoperatively, patients in the repaired TB Group had higher HΗS (Group A, HHS score median: 80, range 62–94; Group B, HHS score median: 84, range 64–96; *P* = 0.052) (Fig. 3) and experienced slightly less LTP when lying on the operative side (Group A, VAS score median: 2, range 0–4; Group B, VAS score median: 1, range 0–3; *P* = 0.046) (Fig. 4a). However, at the same time period, the VAS trochanteric pain scores during activities were similar between groups (Group A, VAS score median: 1, range 0–4; Group B, VAS score median: 1, range 0–4; *P* = 0.759) (Fig. 4b). Thereafter, no differences regarding HHS or LTP were observed.

Subgroup analysis failed to identify any correlation between high offset stems and LTP during activities and lying on the operated side (*P* > 0.05 for all follow-up time-points).

No leg length discrepancy of more than 0.5 cm or Trendeleburg gait pattern were recorded after 12 months postoperatively. Apart from one patient in Group A and one in Group B suffering from asymptomatic DVT and upper respiratory tract infection, respectively, no major systemic or severe local side effects were recorded.

## Discussion

This study demonstrated that repair of the TB during closure of posterior-approach THA may improve early recovery and patients’ ability to lie on the operated side but did not appear to affect the levels of LTP during routine daily activities. This benefit was evident only in the early postoperative period, as after 3 months no clear advantage was identified. The majority of rehabilitation protocols do not support lying on the operated leg for at least 4–6 weeks after surgery. This recommendation is somewhat arbitrary as there is no evidence to support that this position is harmful for patients undergoing posterior-approach THA in the early (< 6 weeks) postoperative period. We encouraged all patients to lie on the operated side from the first week postoperatively, with a pillow between the legs to avoid adduction and stretching of the lateral thigh and buttock soft-tissue structures. In recent years we have abandoned the typical posterior approach in favor of the direct superior approach, which does not violate the trochanteric area, and we have noticed that these patients have considerably less LTP. The direct superior approach also preserves the ITB and keeping this structure intact is likely to contribute to the lower incidence of LTP.

We hypothesize this result is related to the fact that the TB forms a sort of cushion between the greater trochanter and laterally located soft tissues, acting as a “shock absorber” not only throughout joint movement but also during compression caused by lying on the operated side. Furthermore, radiological studies have found abductor tendon abnormalities and peritendinous edema not only after lateral hip approached THA but even in non-operated patients suffering from trochanteric type pain [5, 6]. The increased vascular capacity of the bursa tissue may improve the healing potential of the dissected posterolateral hip structures as well as tendon's regeneration and recovery [1].

Lateral trochanteric pain following THA is a recognized phenomenon and its prevalence may range from 4 to 17% [7–10]. The etiology of LTP has been largely attributed to postsurgical scar tissue formation and development of trochanteric bursitis [11]. However, this theory has not been confirmed by histological studies, which failed to reveal any acute or chronic inflammation of the examined bursae from patients suffering from trochanteric pain in the setting of THA [12–14]. So, the term bursitis may not accurately describe the origin and characteristics of the symptoms, in such cases. Surgical approach seems to be an independent variable, as the incidence of postoperative LTP was found to be higher when a direct lateral approach was applied instead of a posterior approach (4.9% *v*s. 1.2%) [15].

Alteration of hip biomechanics in THA has also been questioned as a cause of lateral hip pain. However, studies have often found inconclusive or underpowered associations between these factors and the development of LTP [16]. It has been postulated that excessive femoral offset, in an effort to improve stability of THA, may cause prominence of the greater trochanter and produce LTP. In addition, leg lengthening may cause tightening of the soft tissues and cause alterations in gait pattern and in the distribution of forces around the hip [17]. Iorio *et al*. did not find any correlation between increased femoral offset or leg length alteration and incidence of LTP after THA [15]. Our study also failed to identify any association between LTP and stem offset. Farmer *et al*. found that patients who needed treatment with cortisone injection due to trochanteric pain after primary THA had greater limb-length inequality [11]. However, they did not perform a comparison between those who developed LTP and those who did not, so no definitive conclusion could be made.

Strengths of this study include its prospective randomized nature, the comparison of multiple functional parameters and the homogeneity of the study population, as the baseline demographics between groups were comparable. An inherent limitation of the study was that the intervention was unblinded, which could create the potential for bias. Also, subgroup analysis results are underpowered; the study’s a priori power analysis was based on HHS, which was one of the two primary objectives. Therefore, the statistical power of LTP results may not be the same as with HHS results.

## Conclusion

Despite the fact that the specific factors predisposing to LTP development are still not well understood, TB repair during posterolateral hip approaches may decrease patient’s discomfort lying on the operated side and improve hip function at the early postoperative period.

## Data Availability

Not Applicable.

## Abbreviations

TB: Trochanteric Bursa
THA: Total Hip Arthroplasty
LTP: Lateral Trochanteric Pain
HHS: Harris Hip Score
VAS: Visual Analogue Scale

## References

[1] Dunn T, Heller CA, McCarthy SW, Dos Remedios C (2003). Anatomical study of the “trochanteric bursa”. Clin Anat.

[2] Pfirrmann CW, Chung CB, Theumann NH, Trudell DJ, Resnick D (2001). Greater trochanter of the hip: attachment of the abductor mechanism and a complex of three bursae--MR imaging and MR bursography in cadavers and MR imaging in asymptomatic volunteers. Radiology.

[3] Woodley SJ, Mercer SR, Nicholson HD (2008). Morphology of the bursae associated with the greater trochanter of the femur. J Bone Joint Surg Am.

[4] Faul F, Erdfelder E, Buchner A, Lang AG (2009). Statistical power analyses using G*power 3.1: tests for correlation and regression analyses. Behav Res Methods.

[5] Klauser AS, Martinoli C, Tagliafico A, Bellmann-Weiler R, Feuchtner GM, Wick M, Jaschke WR (2013). Greater trochanteric pain syndrome. Semin Musculoskelet Radiol.

[6] Steinert L, Zanetti M, Hodler J, Pfirrmann CW, Dora C, Saupe N (2010). Are radiographic trochanteric surface irregularities associated with abductor tendon abnormalities?. Radiology.

[7] Chung CB, Robertson JE, Cho GJ, Vaughan LM, Copp SN, Resnick D (1999). Gluteus medius tendon tears and avulsive injuries in elderly women: imaging findings in six patients. AJR Am J Roentgenol.

[8] Connell DA, Bass C, Sykes CA, Young D, Edwards E (2003). Sonographic evaluation of gluteus medius and minimus tendinopathy. Eur Radiol.

[9] Kingzett-Taylor A, Tirman PF, Feller J, McGann W, Prieto V, Wischer T, Cameron JA, Cvitanic O, Genant HK (1999). Tendinosis and tears of gluteus medius and minimus muscles as a cause of hip pain: MR imaging findings. AJR Am J Roentgenol.

[10] Kong A, Van der Vliet A, Zadow S (2007). MRI and US of gluteal tendinopathy in greater trochanteric pain syndrome. Eur Radiol.

[11] Farmer KW, Jones LC, Brownson KE, Khanuja HS, Hungerford MW (2010). Trochanteric bursitis after total hip arthroplasty: incidence and evaluation of response to treatment. J Arthroplast.

[12] Abdulkarim A, Keegan C, Bajwa R, Sheehan E (2018). Lateral trochanteric pain following total hip arthroplasty: radiographic assessment of altered biomechanics as a potential aetiology. Ir J Med Sci.

[13] Board TN, Hughes SJ, Freemont AJ (2014). Trochanteric bursitis: the last great misnomer. Hip Int.

[14] Silva F, Adams T, Feinstein J, Arroyo RA (2008). Trochanteric bursitis: refuting the myth of inflammation. J Clin Rheumatol.

[15] Iorio R, Healy WL, Warren PD, Appleby D (2006). Lateral trochanteric pain following primary total hip arthroplasty. J Arthroplast.

[16] Capogna BM, Shenoy K, Youm T, Stuchin SA (2017). Tendon disorders after Total hip arthroplasty: evaluation and management. J Arthroplast.

[17] Wretenberg P, Hugo A, Brostrom E (2008). Hip joint load in relation to leg length discrepancy. Med Devices (Auckl).

